# A Patient Similarity Network (CHDmap) to Predict Outcomes After Congenital Heart Surgery: Development and Validation Study

**DOI:** 10.2196/49138

**Published:** 2024-01-19

**Authors:** Haomin Li, Mengying Zhou, Yuhan Sun, Jian Yang, Xian Zeng, Yunxiang Qiu, Yuanyuan Xia, Zhijie Zheng, Jin Yu, Yuqing Feng, Zhuo Shi, Ting Huang, Linhua Tan, Ru Lin, Jianhua Li, Xiangming Fan, Jingjing Ye, Huilong Duan, Shanshan Shi, Qiang Shu

**Affiliations:** 1 Clinical Data Center, The Children’s Hospital, Zhejiang University School of Medicine, National Clinical Research Center for Child Health, Hangzhou, China; 2 The College of Biomedical Engineering and Instrument Science, Zhejiang University, Hangzhou, China; 3 Cardiac Intensive Care Unit, The Children’s Hospital, Zhejiang University School of Medicine, National Clinical Research Center for Child Health, Hangzhou, China; 4 Ultrasonography Department, Zhejiang University School of Medicine, National Clinical Research Center for Child Health, Hangzhou, China; 5 Cardiac Surgery, The Children’s Hospital, Zhejiang University School of Medicine, National Clinical Research Center for Child Health, Hangzhou, China

**Keywords:** medicine-based evidence, general prediction model, patient similarity, congenital heart disease, echocardiography, postoperative complication, similarity network, heart, cardiology, NLP, natural language processing, predict, predictive, prediction, complications, complication, surgery, surgical, postoperative

## Abstract

**Background:**

Although evidence-based medicine proposes personalized care that considers the best evidence, it still fails to address personal treatment in many real clinical scenarios where the complexity of the situation makes none of the available evidence applicable. “Medicine-based evidence” (MBE), in which big data and machine learning techniques are embraced to derive treatment responses from appropriately matched patients in real-world clinical practice, was proposed. However, many challenges remain in translating this conceptual framework into practice.

**Objective:**

This study aimed to technically translate the MBE conceptual framework into practice and evaluate its performance in providing general decision support services for outcomes after congenital heart disease (CHD) surgery.

**Methods:**

Data from 4774 CHD surgeries were collected. A total of 66 indicators and all diagnoses were extracted from each echocardiographic report using natural language processing technology. Combined with some basic clinical and surgical information, the distances between each patient were measured by a series of calculation formulas. Inspired by structure-mapping theory, the fusion of distances between different dimensions can be modulated by clinical experts. In addition to supporting direct analogical reasoning, a machine learning model can be constructed based on similar patients to provide personalized prediction. A user-operable patient similarity network (PSN) of CHD called CHDmap was proposed and developed to provide general decision support services based on the MBE approach.

**Results:**

Using 256 CHD cases, CHDmap was evaluated on 2 different types of postoperative prognostic prediction tasks: a binary classification task to predict postoperative complications and a multiple classification task to predict mechanical ventilation duration. A simple poll of the *k*-most similar patients provided by the PSN can achieve better prediction results than the average performance of 3 clinicians. Constructing logistic regression models for prediction using similar patients obtained from the PSN can further improve the performance of the 2 tasks (best area under the receiver operating characteristic curve=0.810 and 0.926, respectively). With the support of CHDmap, clinicians substantially improved their predictive capabilities.

**Conclusions:**

Without individual optimization, CHDmap demonstrates competitive performance compared to clinical experts. In addition, CHDmap has the advantage of enabling clinicians to use their superior cognitive abilities in conjunction with it to make decisions that are sometimes even superior to those made using artificial intelligence models. The MBE approach can be embraced in clinical practice, and its full potential can be realized.

## Introduction

Congenital heart disease (CHD) is the most common type of birth defect, with birth prevalence reported to be 1% of live births worldwide [1]. Despite remarkable success in the surgical and medical management that has increased the survival of children with CHD [2], the quality of treatment and prognosis after congenital heart surgery remains unsatisfactory and varies across centers [3,4]. The reason for this is that the complexity of the disease, clinical heterogeneity within lesions, and small number of patients with specific forms of CHD severely degrade the precision and value of estimates of average treatment effects provided by randomized controlled trials on the average patient. Some visionary researchers have proposed a new paradigm called “medicine-based evidence” (MBE), in which big data and machine learning techniques are embraced to interrogate treatment responses among appropriately matched patients in real-world clinical practice [5,6].

Postoperative complications in congenital heart surgery have been inconsistently reported but have important contributions to mortality, hospital stay, cost, and quality of life [7-9]. Heart centers with the best outcomes might not report fewer complications but rather have systems in place to recognize and correct complications before deleterious outcomes ensue [8]. The early detection of deterioration after congenital heart surgery enables prompt initiation of therapy, which may result in reduced impairment and earlier rehabilitation. Several risk scoring systems, such as the Risk Adjustment for Congenital Heart Surgery 1 (RACHS-1) method, Aristotle score, and Society of Thoracic Surgeons–European Association for Cardiothoracic Surgery (STS-EACTS) score, have been developed and used to adjust the risk of in-hospital morbidity and mortality [10-13]. However, most of these consensus-based risk models only focus on the procedures themselves and ignore the differences between centers and patients. Specific patient characteristics, such as lower weight [14] and longer cardiopulmonary bypass time [15], especially the quantitative echocardiographic indicators used by clinicians to understand CHD conditions, were not incorporated into these models nor can they be adjusted for. Based on the increasing number of CHD databases being built, some machine learning–based predictive models have recently been used to identify independent risk factors and predict complications after congenital heart surgery [16-18]. These predictive models achieved outstanding performance compared to traditional risk scores, but these models are usually only capable of performing a single task. In addition, such models often contain hundreds of features, so for clinicians, understanding how to interpret the prediction from a complicated machine learning model is still a challenge [19]. Based on our previous studies [16-18], as the model becomes more complex and more variables are included, the results are better, but it is more difficult to understand and accept clinically. Although some explainable artificial intelligence (AI) techniques continue to evolve [20,21], machine learning prediction models are still a black box for clinicians. Due to the lack of understanding and manipulation of the model, clinicians often lack confidence in the predicted outcomes, which severely hampers the entry of these machine learning models into routine care.

Patient similarity networks (PSNs) are an emerging paradigm for precision medicine, in which patients are clustered or classified based on their similarities in various features [22,23]. PSNs address many challenges in data analytics and is naturally interpretable. In a PSN, each node is an individual patient, and the distance (or edge) between 2 nodes corresponds to pairwise patient similarity for given features. PSNs naturally handle heterogeneous data, as any data type can be converted into a similarity network by defining similarity measures [24,25]. A PSN generated based on a large cohort of patients will show several subgroups of patients who are tightly connected. If a new patient is located on the PSN, neighbors that have similar features with known risk or prognosis will inform clinicians of the potential risk and prognosis of the patient. This mimics the clinical reasoning of many experienced clinical experts, who often relate a patient to similar patients they have seen. Moreover, representing patients by similarity is conceptually intuitive and explainable because it can convert the data into network views, where the decision boundary can be visually evident [26]. PSNs can also provide a feasible engineering solution for the MBE framework, which, based on a library of “approximate matches” consisting of a group of patients who share the greatest similarity with the index case, can be examined to estimate the effects of various treatments within the context of the individual patient’s specific characteristics [6].

PSNs have been reported in many studies. Although early PSN studies have focused on using omics data in precision medicine [27-29], with the development of electronic health record (EHR) systems, abundant, complex, high-dimensional, and heterogeneous data are being captured during daily care, and some EHR-based patient similarity frameworks have been proposed for diagnosis [30], subgroup patients [31,32], outcome prediction [33], drug recommendation [34,35], and disease screening [36]. However, studies of PSNs that predict the outcome after CHD surgery have not been reported. A perspective article proposed an MBE conceptual framework for CHD [6], in which similarity analysis is used to generate a library of “approximate matches.” However, they did not provide any technical solution for this framework. The challenge in applying PSNs in a real clinical setting is, first of all, to assess the distance between patients with complex conditions such as CHD in a computable way. However, mimicking clinical analogy reasoning is not a simple math formula based on various patients’ attributes. The structure-mapping theory in cognitive science argues that advanced cognitive functions are involved in the analysis of relationship similarity above attribute similarity [37]. Analogy inference requires advanced cognitive activity, which current AI technology lacks but clinical experts are good at. However, all established models ignore this important feature of patient similarity analysis, in that it should not only measure patients’ distance but also put clinicians back behind the wheel to generate MBE for clinical decision-making. In this study, we aimed to develop and evaluate a clinician-operable PSN of CHD to try to mitigate the above problems.

## Methods

### Study Design and Population

**Figure 1.**
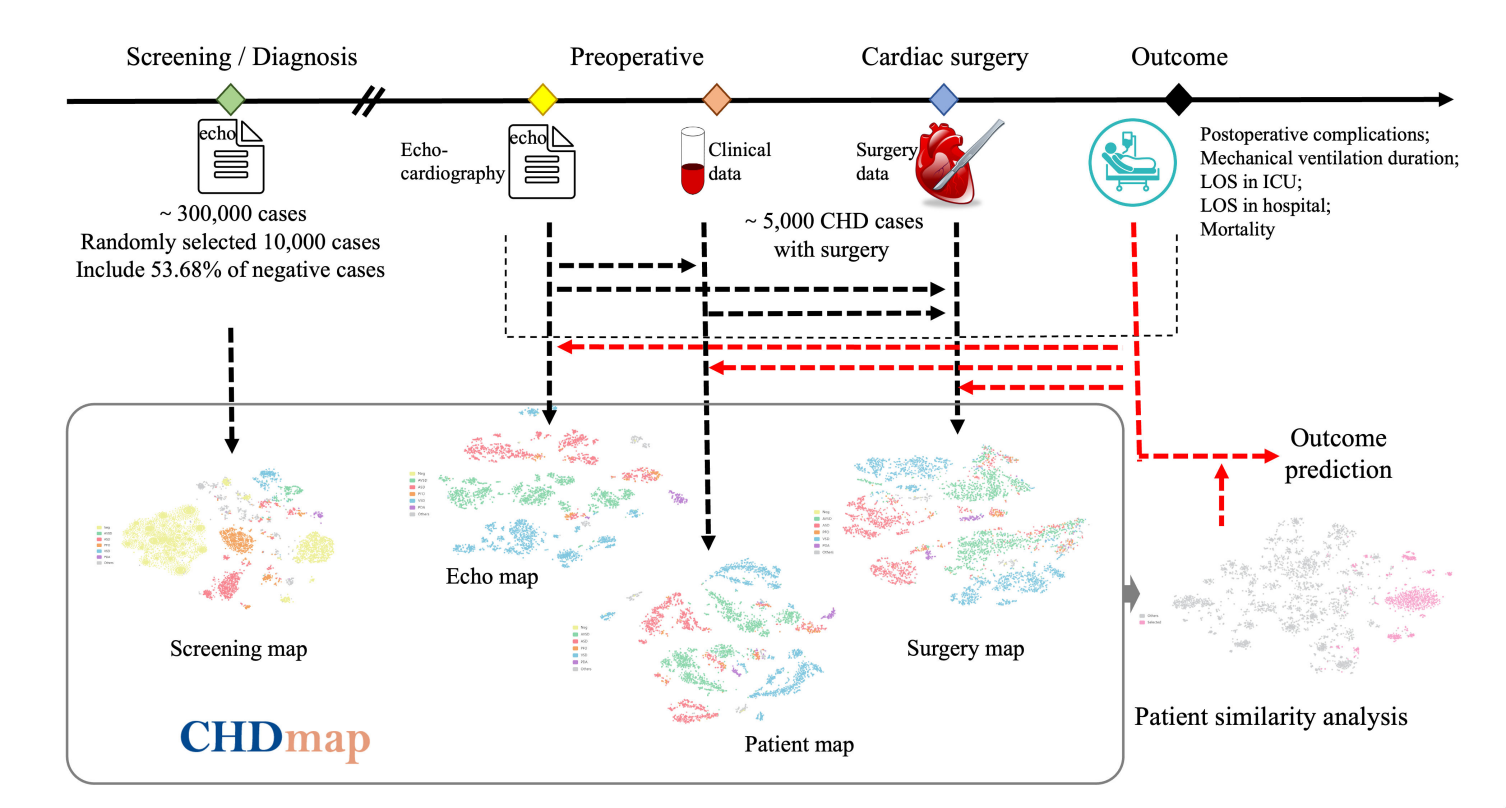
CHDmap contains 4 patient similarity networks generated from 4 different clinical phases, with different data obtained at each phase. CHD: congenital heart disease; ICU: intensive care unit; LOS: length of stay.

As shown in Figure 1, using data available at different stages, 4 PSNs were generated and named as screening map, echo map, patient map, and surgery map. These data were obtained from the ultrasound reporting system and EHR system of the Children’s Hospital, Zhejiang University School of Medicine, Hangzhou, China.

**Figure 2.**
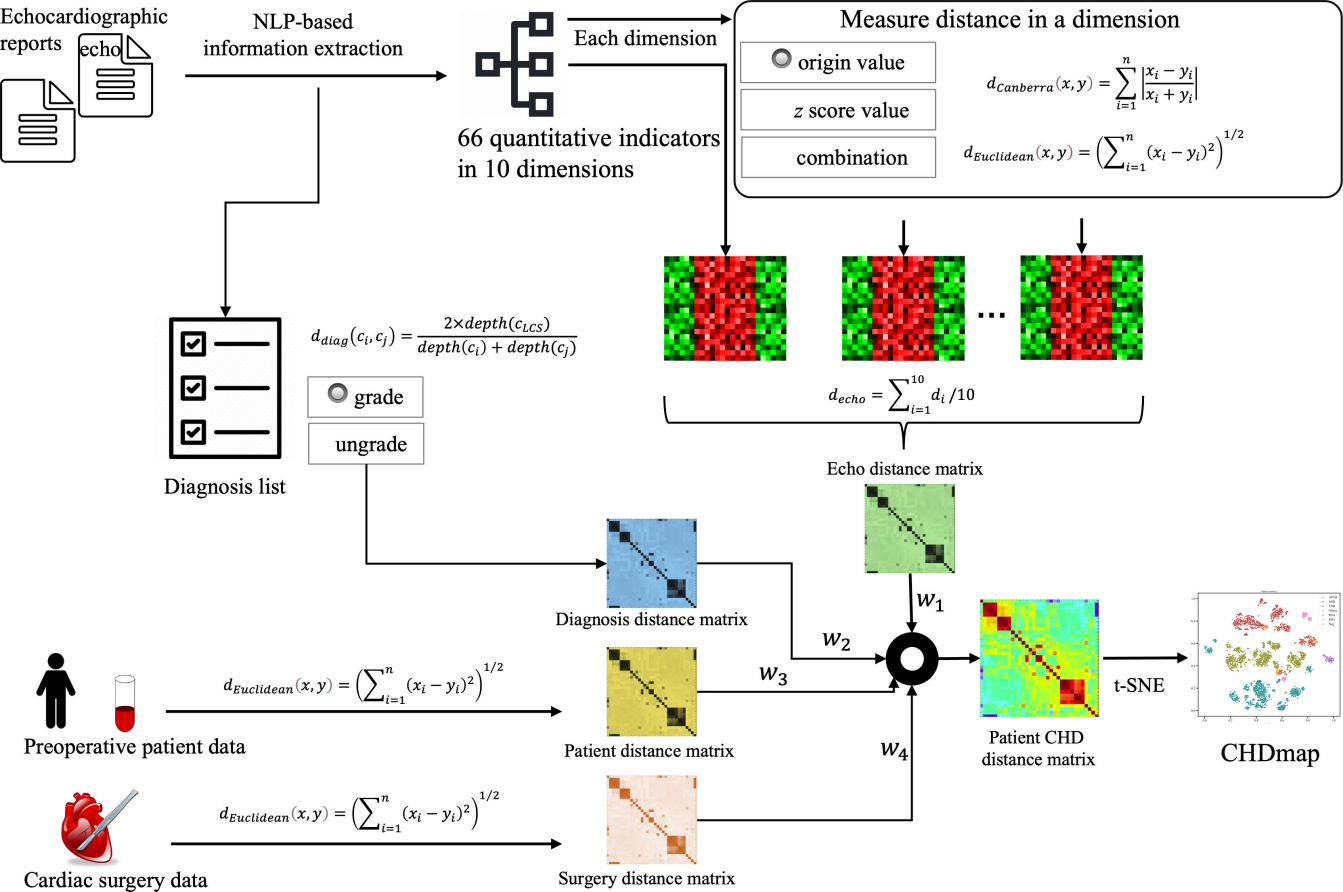
Schematic of data processing and workflow of the construction of the congenital heart disease (CHD) patient similarity network. NLP: natural language processing; t-SNE: t-distributed stochastic neighbor embedding.

A schematic of the data processing and workflow for the construction of the PSN is shown in Figure 2 and described below.

### Ethical Considerations

This retrospective study was performed according to relevant guidelines and approved by the institutional review board of the Children’s Hospital of Zhejiang University School of Medicine with a waiver of informed consent (2018_IRB_078). All cases included in this study were anonymized. Intensive care unit (ICU) clinicians who participated in the trial received cash compensation (RMB ¥100 [US $14.06] per day), which complied with local regulatory requirements for scientific labor.

### Data Collection and Preprocessing

In addition to preoperative echocardiography reports that described the CHD conditions, the following patient and surgical characteristics were also collected: age, sex, height, weight, preoperative oxygen saturation of the right-upper limb, surgery time, cardiopulmonary bypass time, aortic cross-clamping time, mechanical ventilation time, duration of postoperative hospital stay, duration of ICU stay, and postoperative complications (the detailed definitions of postoperative complications are shown in Table S1 in Multimedia Appendix 1 [38-40]).

The most challenging part of patient similarity analysis was defining all the semantic concepts in the domain. An ontology of CHD was developed based on reviewing a large number of clinical guidelines for CHD to cover 436 CHD conditions and 87 related echocardiographic indicators. The OWL format ontology file is available on the CHDmap website [41]. The ontology was used to normalize all concepts and measure semantic similarity among them. It was also used to identify quantitative indicators from the unstructured text of echocardiography reports. In addition to recording some routine cardiac structure indicators, the echocardiography report also provided quantitative indicators regarding various malformations, such as the size of various defects, shunt flow velocity, and pressure difference at the defect, depending on the specific CHD structural malformation. Natural language processing (NLP) technology [38] was used to extract 66 commonly used quantitative indicators. A range of processing and computational methods were used to assess similarity between patients (details information are shown in the supplemental methods and Tables S2 Table S3 in Multimedia Appendix 1). The various automatically extracted measurement values were subject to quality control, and any abnormal data (outside the reasonable range of the corresponding values) were modified or removed after manual verification. The diagnosis in the report was also extracted and mapped to the normalized terms defined in the CHD ontology.

### Measuring Patient Similarity

In this study, the similarity of patients with CHD was measured using 4 groups of features: the quantitative echocardiographic indicators, the specific CHD diagnosis, preoperative clinical features, and surgical features. Different distance measurement methods were adopted for different groups of features, as described in the supplemental methods in Multimedia Appendix 1. We provided 3 types of methods to handle the echocardiographic indicators: the origin value, the *z* score, and the indicator combination ratio. The similarity between 2 diagnoses was calculated using the depth of the corresponding nodes in the CHD ontology, which organizes hundreds of CHD diagnoses in a hierarchical structure. Two approaches were used to measure the distance between diagnosis lists: one treats all diagnoses equally, referred to in the result section as “ungrade,” whereas the other distinguishes between basic and other diagnoses, referred to as “grade.” Finally, the patient distance was measured as the weighted sum of the 4 distances as shown in equation (1), and the final distances were also normalized to [0,1].


 (1) $$d_{patient}=w_{1}\times d_{indicator}+w_{2}\times d_{Diag}+w_{3}\times d_{pre}+w_{4}\times d_{surg}$$

The weights in equation (1) and the different methods used to measure distance can also be modified by users depending on their experience in different tasks to fully exploit the advanced cognitive ability of clinical professionals. The distance matrix among historical patients can be calculated based on the aforementioned methods. We used t-distributed stochastic neighbor embedding [42] to convert the distance matrix into 2D points, which can be visualized as a map. The user-operable CHDmap was developed based on ECharts [43] using React (Meta) and Node.js (OpenJS Foundation). The patient similarity analysis engine, which measures the distances between a new patient and patients in CHDmap, was developed using Python (Python Software Foundation).

### CHDmap

**Figure 3.**
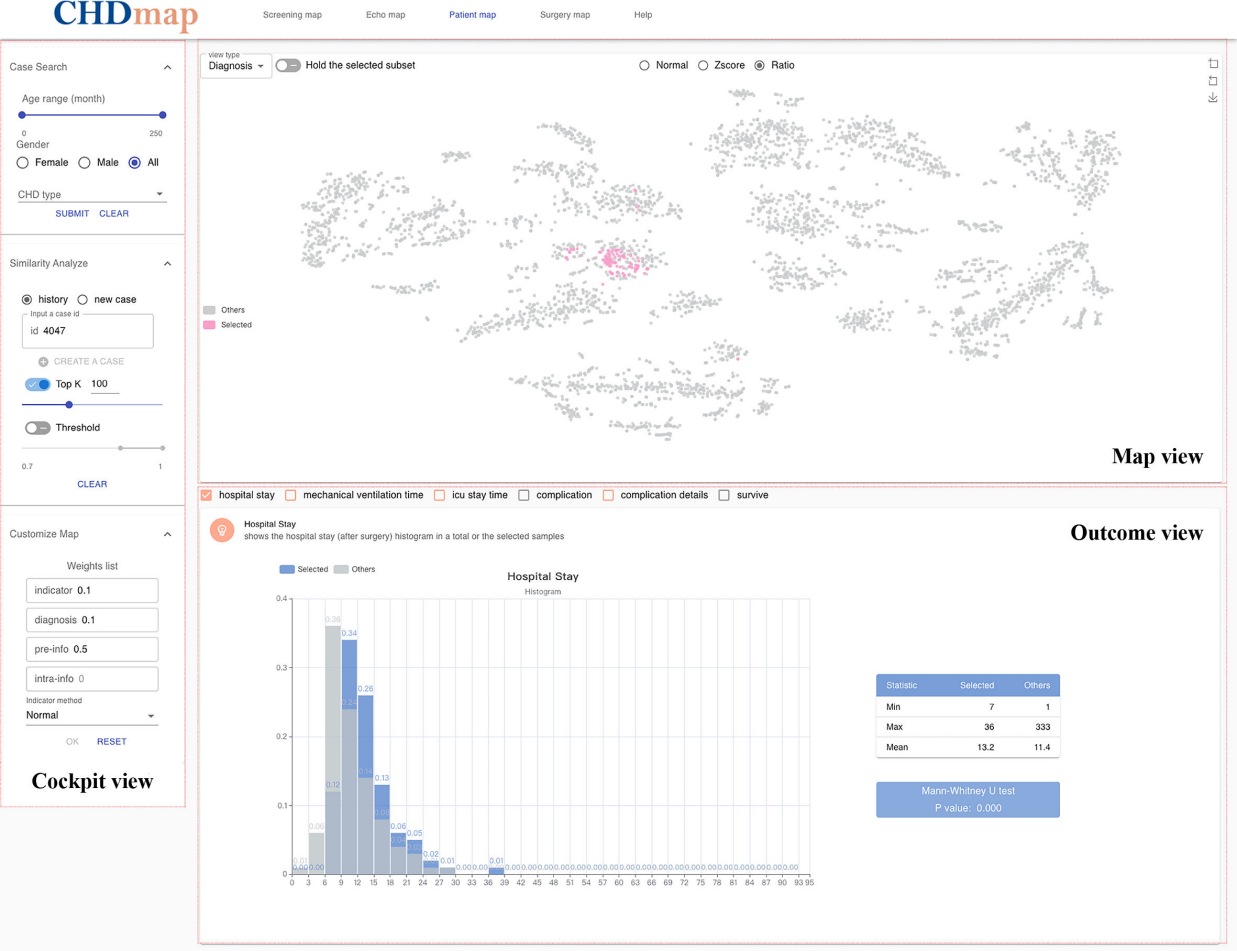
Screenshot of CHDmap. The map view, cockpit view, and outcome view of the workspace are marked separately. CHDmap was published on the web [44]. CHD: congenital heart disease.

A user-operable CHD PSN called CHDmap was developed and published on the web [44]. The introduction video of this tool is also available in Multimedia Appendix 2. Based on the different available data for each clinical phase, as shown in Figure 1, CHDmap provides 4 different PSNs: the screening map, echo map, patient map, and surgery map. The workspace of CHDmap comprises 3 major modules: (1) map view, (2) cockpit view, and (3) outcome view (as shown in Figure 3).

The map view presents the PSN as a zoomable electronic map, in which a node presents a patient and the distance between nodes shows their similarity. The map can be enhanced by using different colors to show the diagnostic labels as well as relevant prognostic indicators (eg, length of stay and complications). Different methods to handle the echocardiographic indicators, such as normal, z score, or combination ratio value, can be selected on the web. The similar patient group is also highlighted on the map view during similarity analysis.

The cockpit view provides a navigation function that helps clinicians locate cases based on specified query conditions, such as age, gender, and CHD subtypes. In practice, clinicians were allowed to create a new case, in which an NLP-based information extraction tool will assist users in filling in most of the echocardiographic indicators based on Chinese echocardiography reports. The top *k* value, or threshold of patient similarity, is used to customize the similar group. For advanced users, a customized map can be generated by adjusting the weights for the patient similarity measurement defined in the *Methods* section.

The outcome view provides an overview of outcomes, including the length of hospital stay, mechanical ventilation time, length of ICU stay, complications, and hospital survival of the selected similar patient group. Multiple charts are used to show the difference between the selected patient group and others. The Mann-Whitney *U* test and the *χ*^2^ test are used to determine the significance of differences between groups. When there are significant differences between the selected patient group and other patients, the color of the check box at the top of the outcome view will turn red; otherwise, it will stay gray. Checking the box will show detailed charts and tables of the outcome. This real-time feedback will help clinicians adjust the parameters in the cockpit view based on the requirements of the scenario for clinical decision-making. Based on a selected group of similar patients, CHDmap provides machine learning models to personalize the prediction of relevant outcome metrics for the current patient. Therefore, for each case, different parameters can be applied and compared to ultimately assess the credibility of the relevant decision support information.

### Evaluation Method

The closer 2 patients are located on the CHDmap, the more similar their conditions and postoperative outcomes are considered to be. When a new patient is admitted to the hospital, historical patients can be divided into similar and nonsimilar groups based on some criteria. There are 2 criteria to define patient similarity groups: one is to use the most similar *k* patients, also known as *k*-nearest neighbor (KNN), to form a patient similarity group, and the other is to define a threshold above which patients form a similarity group. The statistical characteristics or regression value of postoperative outcomes in the similarity group are used to predict the outcomes of the current patient.

In this paper, we evaluated the performance of the surgery map of CHDmap on 2 tasks: predicting postoperative complications as a binary classification task, in which more than 50% of patients in the similarity group with complications were assigned ‘True” for the target patient, and predicting mechanical ventilation duration as a multiple-label classification task (I: 0-12 h, II: 12-24 h, III: 24-48 h, and IV: >48 h), in which the category with the highest proportion in the similarity group was assigned to the target patient.

As the optimum *k* of KNN to form a similarity group for a specific case is always different, the unified population-level optimized *k* on the training data set was used to evaluate CHDmap on the test data set without individual customization. Different data preprocessing methods (original, *z* score, and combination ratio) and whether to distinguish primary diagnoses (grade and ungrade) were tested and compared.

Making decisions may not be straightforward if the outcome of a similar patient group is extremely heterogeneous, whereby a machine learning model based on a similar patient population can provide a more personalized prediction of the relevant prognostic indicators. Although there are numerous machine learning models to choose from, the focus of this study was to demonstrate the advantages of basing the model on similar patient populations, so we chose to use the most conventional and easily understood logistic regression (LR) model. Clinical users obtained a population of similar patients after various parameter adjustments and threshold settings on CHDmap, and the data from this population were used to train an LR model (KNN+LR), which can be accomplished on the web in real time because this population of similar patients is usually not very large. To demonstrate the effect of similar patient populations, we trained another LR model (*k*-Random+LR) based on randomly collected cases of the same size in parallel in the evaluation. We evaluated such approaches and compared the LR models based on *k* similar patients and *k* random patients.

The accuracy, recall, *F*_1_-score, and area under the receiver operating characteristic curve (AUC), which are defined below, were adopted to evaluate the performance of the classification. Accuracy is defined as the total correctly classified example including true positive (TP) and true negative (TN) divided by the total number of classified examples. Recall quantifies the number of correct positive predictions made out of all positive predictions that could have been made. *F*_1_-score is a weighted average of precision and recall. As we know, in precision and recall, there are false positive (FP) and false negative (FN), so *F*_1_-score also considers both of them. AUC provides an aggregate measure of the performance across all possible classification thresholds. The higher the accuracy, recall, *F*_1_-score, and AUC, the better the model’s performance is at distinguishing between the positive and negative classes.


 (2) $$Accuracy=\frac{TP+TN}{TP+TN+FP+FN}$$

(3) $$Recall=\frac{TP}{TP+FN}$$

(4) $$Precision=\frac{TP}{TP+FP}$$

(5) $$F_{1}\text{-}score=\;\frac{2\times \left(Recall\times Precision\right)}{\left(Recall+Precision\right)}$$

The performance was evaluated on an independent test set, which included 256 patients with CHD. These test cases were also available on CHDmap when users created a new case. Three clinicians working in the cardiac ICU with extensive experience were also asked to make relevant judgments for these test cases based on their clinical experience. After half a year following the initial trial, we conducted an experiment where the 3 clinicians were asked to make further predictions based on the output of CHDmap, and this prediction was compared with the previous results based on clinical experience alone to validate the benefits of CHDmap in supporting clinical decision-making.

## Results

### Population Characteristics

A total of 4774 patients who underwent congenital heart surgery between June 2016 and June 2021 at the Children’s Hospital of Zhejiang University School of Medicine were used to generate the CHD PSN. The performance of the PSN in predicting complications and mechanical ventilation duration was evaluated on an independent test data set, which included 256 pediatric patients who underwent congenital heart surgery between July 2021 and November 2021 at the Children’s Hospital of Zhejiang University School of Medicine. The characteristics of patients used to generate the PSN and for evaluation are described in Table 1. Since the test data and the data used by the PSN were generated and collected in different time periods, as shown in Table 1, they are somewhat statistically different. The test data were older; therefore, the patients were significantly larger in terms of height and weight (*P*<.001), and there were also relatively large differences in the distribution of outcomes, lower complication rates, and shorter duration of mechanical ventilation. It should be noted that the diagnostic label is not the complete diagnostic information; we just use a few of the most common CHD subtypes to facilitate statistics and visualization, and this cohort contains a complete range of epidemiological characteristics as well as a variety of complex CHD subtypes such as transposition of the great arteries, tetralogy of Fallot, etc, which may appear in various diagnostic labels that they are combined with. When the case has 2 common CHD subtypes, such as ventricular septal defect and patent ductus arteriosus, only the more common subtype, ventricular septal defect, is labeled.

**Table 1.** Characteristics of patients with CHD^a^ used to generate CHDmap and in the test data set.

Characteristic		Patients of CHDmap (n=4774)	Patients of the test data set (n=256)	*P* value
Gender (male), n (%)		2336 (48.9)	111 (43.4)	.09
Age (mo), median (IQR)		12.0 (4.0-32.0)	22.1 (7.8-50.9)	<.001
Height (cm), median (IQR)		75.0 (63.0-94.0)	85.5 (67.0-106.3)	<.001
Weight (kg), median (IQR)		9.2 (6.0-13.4)	10.8 (6.8-16.5)	<.001
Preoperative oxygen saturation (%), median (IQR)		98.0 (97.0-99.0)	98.0 (97.0-99.0)	.007
Surgery time (min), median (IQR)		119.0 (96.0-147.0)	120.0 (100.0-147.0)	.25
Cardiopulmonary bypass time (min), median (IQR)		60.0 (48.0-82.0)	61.5 (49.3-80.0)	.55
Aortic cross-clamping time (min), median (IQR)		40.0 (28.0-54.0)	38.5 (27.0-52.0)	.55
Duration of hospital stay (d), median (IQR)		9.0 (7.0-13.0)	7.0 (6.0-11.0)	.003
Duration of ICU^b^ stay (d), median (IQR)		3.0 (1.0-4.0)	3.0 (1.0-4.0)	.49
**Diagnostic label, n (%)**				.46
	ASD^c^ and VSD^d^	1659 (34.8)	78 (30.5)	
	VSD	1522 (31.9)	94 (36.7)	
	ASD	1228 (25.7)	65 (25.4)	
	PFO^e^	134 (2.8)	5 (2)	
	PDA^f^	123 (2.6)	9 (3.5)	
	Others	108 (2.3)	5 (2)	
**Mechanical ventilation time (%), n (%)**				.001
	I (<12 h)	3009 (63.0)	180 (70.3)	
	II (12-24 h)	918 (19.2)	54 (21.1)	
	III (24-48 h)	433 (9.1)	7 (2.7)	
	IV (≥48 h)	414 (8.7)	15 (5.9)	
Complication, n (%)		1229 (25.7)	48 (18.8)	.02

a CHD: congenital heart disease.

b ICU: intensive care unit.

c ASD: atrial septal defect.

d VSD: ventricular septal defect.

e PFO: patent foramen ovale.

f PDA: patent ductus arteriosus.

### Performance of CHDmap

**Figure 4.**
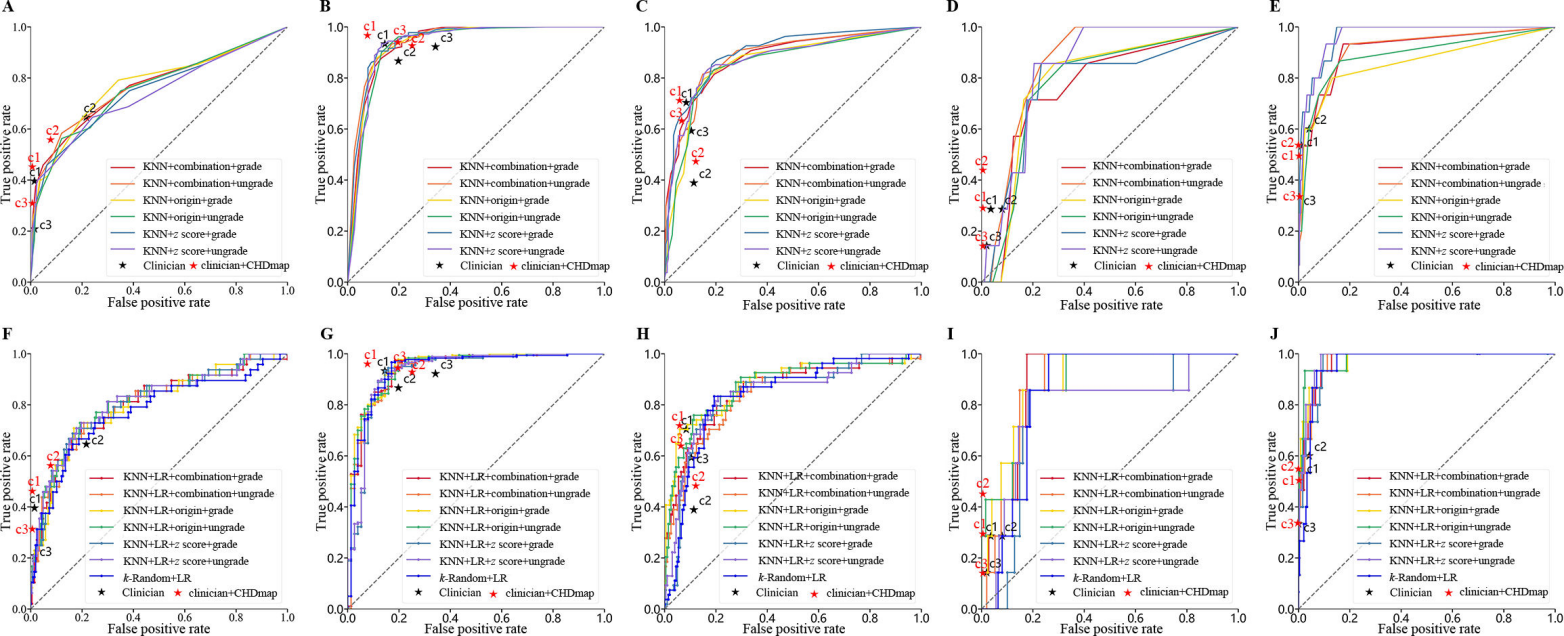
Evaluation result based on receiver operating characteristic curves. (A) Binary postoperative complication prediction using KNN; (B) to (E) multilabel mechanical ventilation duration prediction (I: 0-12 h, II: 12-24 h, III: 24-48 h, and IV: >48 h) using KNN, respectively; (F) binary postoperative complication prediction using KNN+LR; (G) to (J) multilabel mechanical ventilation duration prediction (I: 0-12 h, II: 12-24 h, III: 24-48 h, and IV: >48 h) using KNN+LR, respectively. The performance of 3 clinicians are labeled as black stars in different tasks as C1, C2, and C3. The performance of 3 clinicians enhanced by CHDmap are labeled as red stars. CHD: congenital heart disease; KNN: *k*-nearest neighbor; LR: logistic regression.

Three methods for preprocessing the echocardiographic indicators (origin, *z* score, combination) and 2 distinguishing primary diagnoses (grade and ungrade) were used to compare their effect on CHDmap performance. The performance of the CHDmap and 3 clinicians is shown in Table 2 and Figure 4.

**Table 2.** Evaluation results in the 2 tasks.

Methods		Prediction of postoperative complications				Prediction of mechanical ventilation duration			
		Accuracy	Recall	*F*_1_-score	AUC^a^	Accuracy	Recall	*F*_1_-score	AUC
**KNN^b^**									
	Origin+ungrade	0.832	0.438	0.494	0.757	0.813	0.444	0.459	0.862
	Origin+grade	0.836	0.417	0.489	0.773	0.797	0.437	0.467	0.860
	*z* score+ungrade	0.828	0.458	0.500	0.738	0.836	0.554	0.574	0.902
	*z* score+grade	0.848	0.458	0.530	0.747	0.855	0.564	0.573	0.895
	Combination+ungrade	0.836	0.500	0.533	0.767	0.828	0.468	0.488	0.900
	Combination+grade	0.859	0.458	0.550	0.768	0.855	0.521	0.545	0.873
**KNN+LR^c^**									
	Origin+ungrade	0.813	0.604	0.547	*0.810^d^*	0.848	0.558	0.602	0.921
	Origin+grade	0.813	*0.667*	0.571	0.799	0.863	0.589	0.632	0.920
	*z* score+ungrade	0.809	0.604	0.542	0.809	0.840	0.537	0.561	0.888
	*z* score+grade	0.813	0.646	0.564	0.805	0.855	0.549	0.562	0.886
	Combination+ungrade	0.805	0.583	0.528	0.801	0.840	0.537	0.555	0.900
	Combination+grade	0.805	0.604	0.537	0.798	0.824	0.500	0.522	*0.926*
*k*-Random+LR		0.809	0.500	0.495	0.774	0.809	0.484	0.488	0.895
**Clinicians^e^**									
	C1	0.875	0.396	0.543	N/A^f^	0.844	*0.614*	0.618	N/A
	C2	0.758	0.646	0.500	N/A	0.734	0.535	0.496	N/A
	C3	0.840	0.208	0.328	N/A	0.797	0.498	0.536	N/A
	Clinician average	0.824	0.417	0.457	N/A	0.792	0.549	0.550	N/A
	C1+CHDmap	*0.883*	0.426	*0.580*	N/A	*0.943*	0.612	*0.647*	N/A
	C2+CHDmap	0.816	0.5625	0.534	N/A	0.874	0.587	0.542	N/A
	C3+CHDmap	0.852	0.313	0.441	N/A	0.916	0.511	0.546	N/A
	Clinician+CHDmap average	0.850	0.434	0.518	N/A	0.911	0.570	0.578	N/A

a AUC: area under the receiver operating characteristic curve.

b KNN: *k*-nearest neighbor.

c LR: logistic regression.

d In each column, the maximum value is italicized.

e The performance of the 3 clinicians are labeled as C1, C2, and C3.

f N/A: not applicable.

In the postoperative complication prediction task, the *F*_1_-score of methods using KNN exceeded the average of the 3 clinicians, although 1 clinician achieved the best accuracy when dropping a high recall value. In all 6 KNN methods, introducing the indicator combination ratio and distinguishing the primary diagnosis in the similarity measurement can truly improve the overall performance of the *F*_1_-score. LR models constructed using the KNN-obtained patient groups were able to generally achieve better predictions compared to simple voting of similar patients and the LR model based on *k* random patients. Interestingly, both the model with the best *F*_1_-score performance and the model with the best AUC used the original values. This may be because original values are more reflective of individualized patient differences in a similar patient population. The main improvement of CHDmap on this task is reflected in the general improvement in recall values, with the best recall method being 0.250 higher than the clinician average.

In another multiclassification task that predicts mechanical ventilation duration, the differences among these different KNN methods in overall performance were not consistent. The KNN+LR approaches also achieved better composite performance (*F*_1_-score and AUC), although 1 of the human experts got the best recall value.

From the test result, clinicians do not have the same performance for such predictive judgments. Some raise the standard and thus miss some events; on the other hand, some lower the judgment threshold, and thus the accuracy of the judgment decreases. At the same time, the performance of clinical experts on different tasks is inconsistent. A simple poll of the *k*-most similar patients provided by the CHDmap can achieve better results than the clinician average. When 3 clinicians were allowed to use the results of CHDmap (KNN+LR) as a reference to give predictions again, all 3 clinicians achieved a substantial improvement in their prediction ability. The averages of accuracy, recall, and *F*_1_-score in the first task improved by 0.026, 0.017, and 0.061, respectively. The averages of accuracy, recall, and *F*_1_-score in the second task improved by 0.119, 0.021, and 0.028, respectively. One of the enhanced clinicians also surpassed the KNN+LR CHDmap.

It is important to note that the evaluation is performed with population-optimized parameters, whereas in practice, clinicians can adjust the relevant parameters such as *k* or similarity threshold for each case in a personalized manner, which theoretically leads to better results. The use of the obtained similar patient population to construct modern deep learning models for prediction can further improve the performance of each prediction task. Especially important is that the experience and cognitive ability of the clinical expert combined with CHDmap can further enhance the accuracy of the prediction.

## Discussion

### Principal Findings

Medicine remains both an art and a science, which are congruent to the extent that the individual patient resembles the average subject in randomized controlled trials. Although the evidence-based medicine approach proposes personalized care, it still fails to address the physician’s most important question—“How to treat the unique patient in front of me?”—in many real clinical scenarios where the complexity of the situation makes none of the available evidence applicable [45]. The proposal of MBE represents a fundamental change in clinical decision-making [5,6]. Although how to construct an MBE clinical decision support tool still faces many challenges, the CHDmap seems to be a very promising first step in realizing what has been coined MBE.

AI is poised to reshape health care. Many AI applications, especially modern deep learning models, have been developed in recent years to improve clinical prediction abilities. In addition to supervised and unsupervised machine learning, PSNs, another form of data-driven AI, have shown many unique properties in the clinical field, especially in complex clinical settings such as surgery for CHD. Moreover, their potential to construct a “library of clinical experience” will gradually be recognized, discovered, and used in the context of the continuous accumulation of medical big data.

In many other popular AI paradigms, such as supervised or unsupervised machine learning, models are usually trained toward a specific task, and thus, the models are only capable of performing that single task. This, coupled with the black-box nature of many machine learning models, especially deep learning models, makes it difficult to widely apply these techniques in practice. In contrast, patient similarity analysis exhibits many natural advantages. First, PSNs usually do not serve a single task; all characteristics exhibited by the patient similarity group, such as disease risk, various prognostic outcomes, and cost of care, can be used as MBE for decision support. Second, instead of a model that simply gives black-box predictions, CHDmap allows users to see how the patient similarity group is segmented and bounded across the patient population and then adjust the size of the patient similarity group or set custom quantitative thresholds based on their knowledge and experience. On CHDmap, the results after parameter adjustments during user manipulation are reflected in the visualized map in real time, and the statistical characteristics of multiple predictors that distinguish the current patient’s similar group from other patients are also highlighted by the color of the title of the outcome view. The process of continuously adjusting and optimizing parameters through visualized feedback combines the computational advantages of computers and the advanced cognitive abilities of the human brain and truly puts the clinician, who is responsible for the decision, in control of the decision-making. Third, many machine learning models tend to require that the test and training data have consistent statistical distribution characteristics, but as shown in this evaluation, similarity analyses are still very compatible with test data with different characteristics. Finally, this PSN framework does not exclude any type of machine learning models, and all models constructed based on similar patient populations are expected to be more adaptable to individualized decision-making needs than models trained on heterogeneous populations.

**Figure 5.**
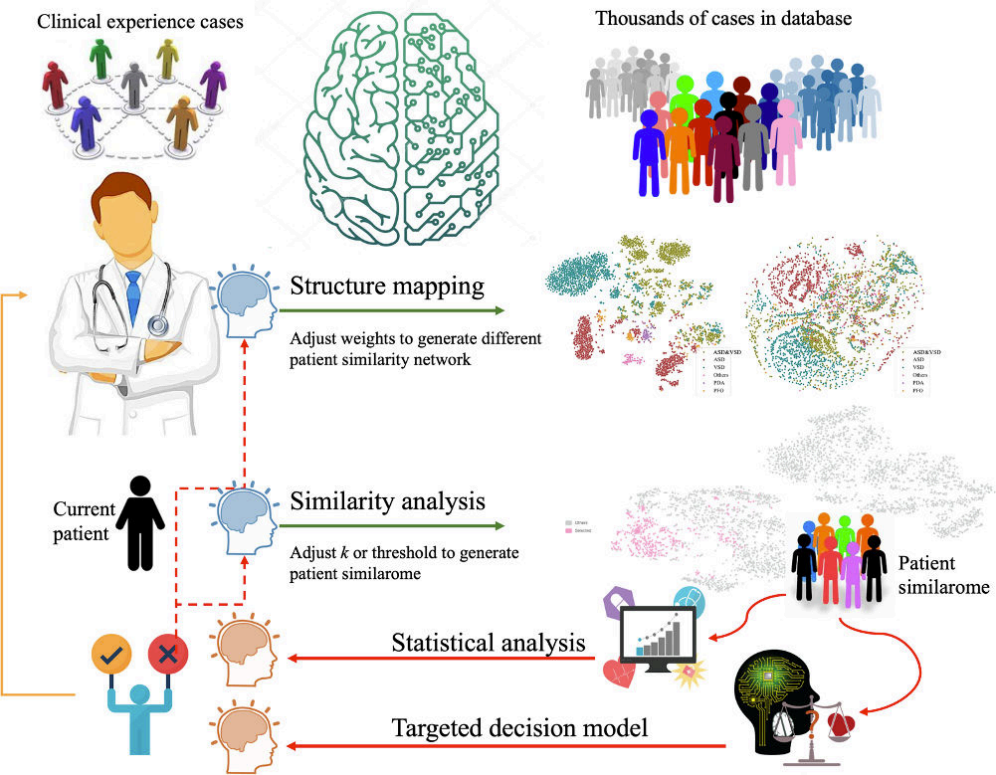
Collaborative decision-making based on the congenital heart disease patient similarity network (PSN). The right half shows the storage and computational capacity of the PSN for a large number of cases; the left half shows the role of the clinical user who, by receiving a variety of feedback and his or her own experience, can autonomously adjust the parameters of the similarity group and reconstruct the similarity network so that the strengths of both can be used to make collaborative decisions. ASD: atrial septal defect; PDA: patent ductus arteriosus; PFO: patent foramen ovale; VSD: ventricular septal defect.

Because the goal of patient similarity analysis is to be able to mimic clinical analogy reasoning, the major challenge is constructing computational patient similarity measurements that are consistent with sophisticated clinical reasoning. This is especially true when faced with complex scenarios containing a large number of dynamic features with different dimensions. Some deep learning models have been introduced to address this challenge [46-49], but they do not exhibit the interpretability and tractability of PSNs. Another way to address this challenge is to open up the computational process to clinicians, allowing them to determine and adjust the weights of different dimensions and thresholds for the similarity group themselves, thus better simulating their clinical reasoning process, as shown in Figure 5. We believe that clinical users will be able to learn how to better optimize these parameters as they continue to gain experience and understanding of this “large history data set” in the process of using CHDmap. Using a data-driven approach on how to customize the parameters of PSNs to be able to self-optimize and adapt to different tasks is also a good research direction for the future. In this study, CHDmap serves as a personalized decision aid for clinicians, using the computer’s power in data storage and processing while giving clinicians more control over the decision-making process. We believe CHDmap can perform better with the full involvement of clinicians.

CHDmap can be used in several scenarios: for the intensivists in cardiac ICUs, CHDmap can be used to predict postoperative complications after cardiac surgery, as evaluated in this paper; for surgeons, CHDmap can also be used to assess the prognosis of surgical procedures; and for departmental managers, CHDmap can be used to assess the lengths of stay and costs. By far, CHDmap is still in the early stages of a research project. Transforming this tool into routine care is dependent on the availability of funding and the willingness of users to change their existing working patterns. The publication of this paper will also facilitate the advancement of our subsequent translational work.

It is important to note that associations between treatments and outcomes obtained by observation in similar patient populations may not be causal. The real causal effects often rely on a matching process to control for the bias introduced by the treatment itself in the selection of patients [50]. An initial demo feature is available on CHDmap to estimate treatment outcome effects based on matched patient groups. CHDmap can match 1 or *k* patients for each patient receiving the treatment using a PSN and then allow for a more visual and unbiased assessment of treatment outcomes by showing the difference in prognosis between these 2 groups of patients. It is important to note that this causal assessment assumes that there are no other factors outside the variables covered by the patient’s similarity analysis that may influence treatment choice or prognosis. Thus, the reliability of this real world–generated evidence usually relies on clinical experts to judge it as well. In future versions, we hope to incorporate more modern frameworks for causal inference (such as DoWhy [51]) to automatically quantitatively assess causal effects as well as their reliability.

There are several limitations to this study. First, limited clinical features were used to measure the similarity of patients with CHD. In addition to the information presented by the echocardiography, there is a wealth of other clinical information that can be used to assess the patient’s status. Second, the use of NLP to automatically extract measurement information can also be subject to errors or mismatches, and although manual quality control is carried out, it is still not possible to ensure that all of the measurements are 100% accurate. Third, just as clinicians gain clinical experience by continuously treating different patients, PSNs need to expand their ability to dynamically accumulate cases. A PSN with a web-based automatic update mechanism will be the next key research step. Fourth, data from only a single center were used to evaluate this tool, and the introduction of data from multiple centers during PSN construction may pose unknown risks that require attention in future studies. Finally, different clinicians may have different decision-making philosophies, and different weights can be assigned to different indicators for different tasks. CHDmap offers only a limited number of customizations that may be difficult to adapt to all scenarios. A way to attribute weights to each of the indicators and dimensions by AI for specific tasks may potentially improve the performance of CHDmap in the future.

### Conclusions

A clinician-operable PSN for CHD was proposed and developed to help clinicians make decisions based on thousands of previous surgery cases. Without individual optimization, CHDmap can obtain competitive performance compared to clinical experts. Statistical analysis of data based on patient similarity groups is intuitive and clear to clinicians, whereas the operable, visual user interface puts clinicians in real control of decision-making. Clinicians supported by CHDmap can make better decisions than both pure experience-based decisions and AI model output results. Such a PSN-based framework can become a routine method of CHD case management and use. The MBE can be embraced in clinical practice, and its full potential can be realized.

## Supplementary material

10.2196/49138 Multimedia Appendix 1 Supplemental methods, definitions of postoperative complications, features used to measure patient similarity, and echocardiographic indicators used in different calculations.

10.2196/49138 Multimedia Appendix 2 Video introduction for CHDmap.

## Abbreviations

AI: artificial intelligence
AUC: area under the receiver operating characteristic curve
CHD: congenital heart disease
EHR: electronic health record
FN: false negative
FP: false positive
ICU: intensive care unit
KNN: *k*-nearest neighbor
LR: logistic regression
MBE: medicine-based evidence
NLP: natural language processing
PSN: patient similarity network
RACHS-1: Risk Adjustment for Congenital Heart Surgery 1
STS-EACTS: Society of Thoracic Surgeons–European Association for Cardiothoracic Surgery
TN: true negative
TP: true positive
