# Supplementary material for: A Patient Similarity Network (CHDmap) to Predict Outcomes After Congenital Heart Surgery: Development and Validation Study
Source: JMIR Med Inform. 2024 Jan 19;12:e49138. doi: 10.2196/49138 (PMC10850852; doi:10.2196/49138)
Supplement: Multimedia Appendix 1 [file medinform-v12-e49138-s001.docx]

**Multimedia Appendix 1**

**Supplemental Methods**

**NLP methods for information extraction**

In this project, NLP technology was used in two places. In the data preprocessing stage, NLP was used to extract 66 commonly used quantitative indicators’ values from thousands of echocardiography reports. In the application, NLP is used to assist the user in quickly entering various types of structured information about the current patient. The original NLP method has been reported in previous study [38]. In this study, we have updated the terms defined by our CHD ontology. In the data preprocessing, a total of 46759 indicators were extracted and only 376 outlier values were identified and manually reviewed. It is basically verified that this NLP system is reliable for extracting this type of information, and therefore we allow the user to complete the information entry by providing a free-text echocardiography report when entering a new patient. However, it still requires the user to confirm that the data extracted by NLP is correct, while allowing the user to manually complete structured data entry.

**Measuring patient similarity**

In this study, CHD patient similarity was measured using four groups of features: the quantitative echocardiographic indicators, the specific CHD diagnosis, preoperative clinical features, and surgical features. Different distance measurement methods were adopted for different groups of features, as described below.

*Measurement of distance between quantitative echocardiographic indicators*

We divided the 66 echocardiographic indicators into 10 dimensions based on their physiological relevance (detail information is shown in Table S2), and for each dimension, a Canberra distance was computed as follows:

$d_{Canberra}\left( x,y \right)=\sum_{i=1}^{n} \left| \frac{x_{i}-y_{i}}{x_{i}+y_{i}} \right|$ *Formula (1)*

Where $x_{i}$ and $y_{i}$ represent the same indicator in two patients, and $n$ indicates the number of indicators in a dimension. The Canberra distance can handle indicators with different value scales. The mean value of the distance of the ten dimensions was used to measure the distance between quantitative echocardiographic indicators.

However, the clinical meaning of the value of indicators is affected by body size, age, sex, race, and ethnicity. The Z scores, which represent the number of standard deviations from the normal range of the indicator, have been widely used in the domain to overcome this effect due to individual differences. We provided another method to measure distance based on Z scores. The Z scores of indicators were computed using the formula proposed by Lopez et al [39] before calculating the distance.

$Z=\frac{[\left( \frac{indicator}{{BSA}^{\alpha}} \right)-(mean value of indexed indicator)]}{SD of indexed indicator}$ *Formula (2)*

where the exponent $\alpha$ is a specific parameter for an indicator, SD is the standard deviation, and BSA represents the body surface area, which is estimated using weight and height. Then, the Euclidean distance of indicators in each dimension is computed as follows:

$d_{Euclidean}\left( x,y \right)={(\sum_{i=1}^{n} {(x_{i}-y_{i})}^{2})}^{1/2}$ *Formula (3)*

Considering that the Z score was not always available, we explored an alternative solution using combinations of indicators (e.g., VSD size/diameter of aorta) to reflect the extent to which the indicators deviate from normal values. The correlations of a single indicator and an indicator ratio combination with outcomes were studied in a previous study [40]. Indicator combinations with high correlation (shown in Table S3) were used in the third method and calculated the Canberra distance for each dimension.

*Measurement of the distance between diagnosis list*

The distance between two diagnoses is calculated using the depth of the corresponding nodes in the CHD ontology, which organize hundreds of CHD diagnoses in a hierarchical structure:

$d_{d}\left( c_{i},c_{j} \right)=1-\frac{2\times depth(c_{LCS})}{depth\left( c_{i} \right)+depth(c_{j})}$ *Formula (4)*

where $c_{i}$ and $c_{j}$ represent two nodes in the CHD ontology, respectively, and $c_{LCS}$ is the minimum common ancestor of $c_{i}$ and $c_{j}$. The $depth(c)$ represents the number of nodes on the shortest path from this node to the root node.

Because a patient may have more than one diagnosis, the diagnosis list distance is calculated in two ways. The first is to calculate the mean of the minimum distance in both directions as follows:

$d_{Diag}(A,B)=\frac{1}{m+n}(\sum_{1\leq i\leq m} \min_{1\leq j\leq n} d_{d}\left( a_{i},b_{j} \right)+\sum_{1\leq j\leq n} \min_{1\leq i\leq m} d_{d}\left( b_{j}{,a}_{i} \right))$ *Formula (5)*

where $A$ and $B$ represent two patients, $A$ has m diagnoses $[a_{1},a_{2},\ldots a_{m}]$, and $B$ has n diagnoses $[b_{1},b_{2},\ldots b_{n}]$.The second method requires distinguishing between the diagnoses in the diagnostic list as primary and secondary diagnoses. First, we calculate the mean of the minimum distance in both directions of the primary diagnosis and the secondary diagnosis separately and then calculate the weighted sum of the two.

*Measurement of the distance between patient*

Several patient preoperative features and surgical features were also used to measure patient similarity. As numeric types, they can be calculated directly using Euclidean distances separately to obtain $d_{pre}$ and $d_{surg}$. Finally, the patient distance was measured as the weighted sum of the four distances as shown in Formula 6and the final distances were also normalized to [0,1].

$d_{patient}=d_{echo indicator}\times w_{1}+d_{Diag}\times w_{2}+d_{pre}\times w_{3}+d_{surg}\times w_{4}$ *Formula (6)*

To give full play to the advanced cognitive ability of clinical experts, the weights in formula (6) and different methods to measure distance can also be customized by users based on their experience in different tasks.

**Case Study**

As shown in Figure S1a, the N.O. 225 case is a female infant weighting only 2.9 kg with TGA, ASD, PDA, and tricuspid regurgitation. The “Top 100” criterion was set in Figure S1a, and “Top 30” criterion was set in Figure S1b. The map views show different selected patients under these criteria.


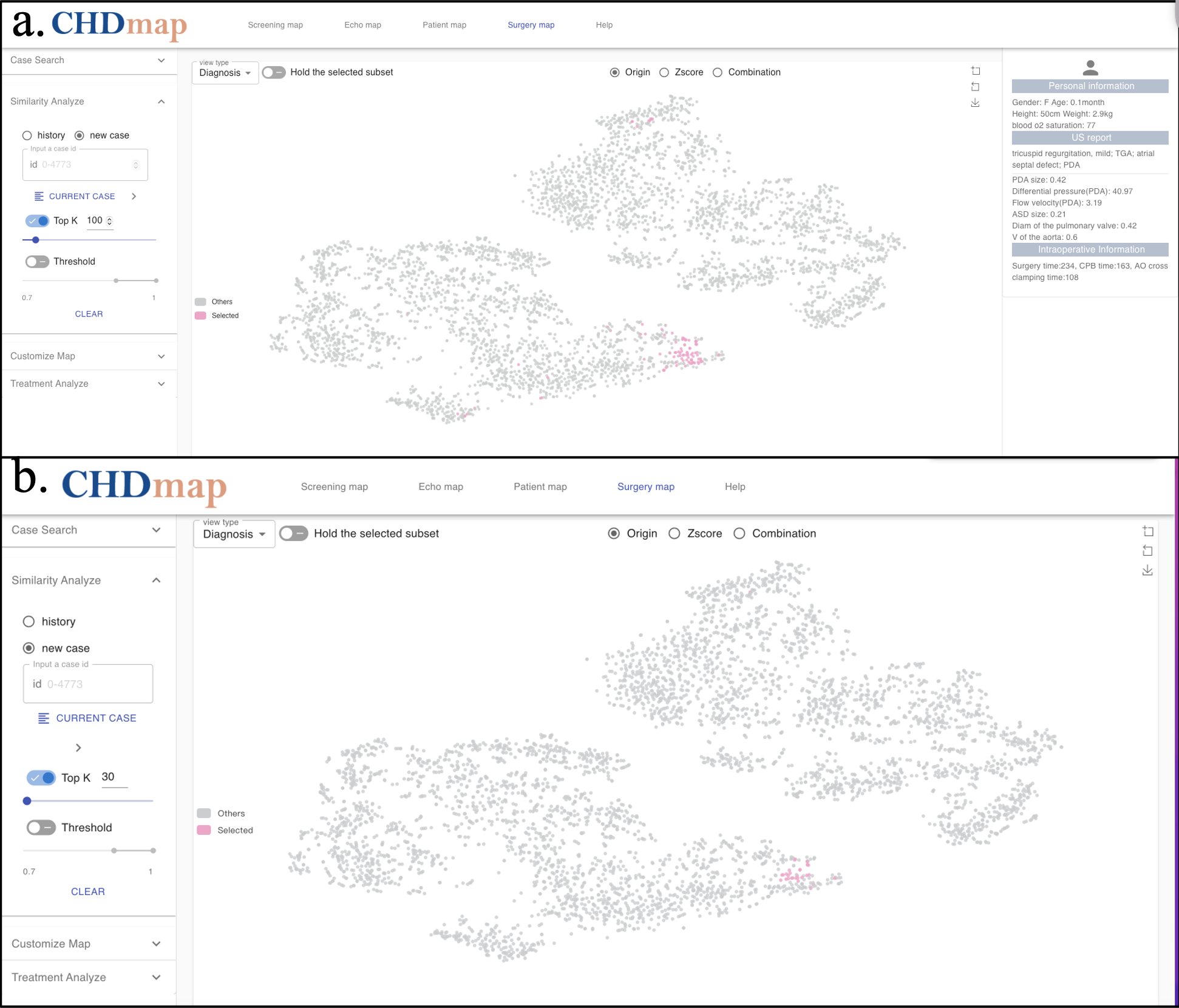


Figure S1 The screenshot of similarity patients of N.O. 225 case in the test dataset in CHDmap. (a). The top 100 patients were selected. (b). The top 30 patients were selected.

A smaller similarity group guarantees a more homogeneous similarity group, while a large relative group guarantees the predictive power of the data. It is often necessary for the user to adjust this threshold based on the proportion of specific CHD subtypes in the historical data. By hovering the mouse over a specific patient node, the user can browse the information about this patient to further determine if the threshold for this similarity analysis is appropriate. In this case, as the total TGA patients in this population is about 80, the criterion of the “Top 100” seems to be a bit large and would incorporate some other CHD subtypes of cases; in the evaluation in this paper, we did not make individualized parameter adjustments.

In the outcome views shown in Figure S2-S5, the characters of the 100 selected matched patients were shown:

**Hospital Stay:** The length of stay in the selected group of similar patients was significantly longer in the distribution, with a mean value of 21.4 days, compared to a mean value of only 11.2 days for the other patients. The use of the prediction tool showed a 97% probability that they would be hospitalized for more than 13 days.


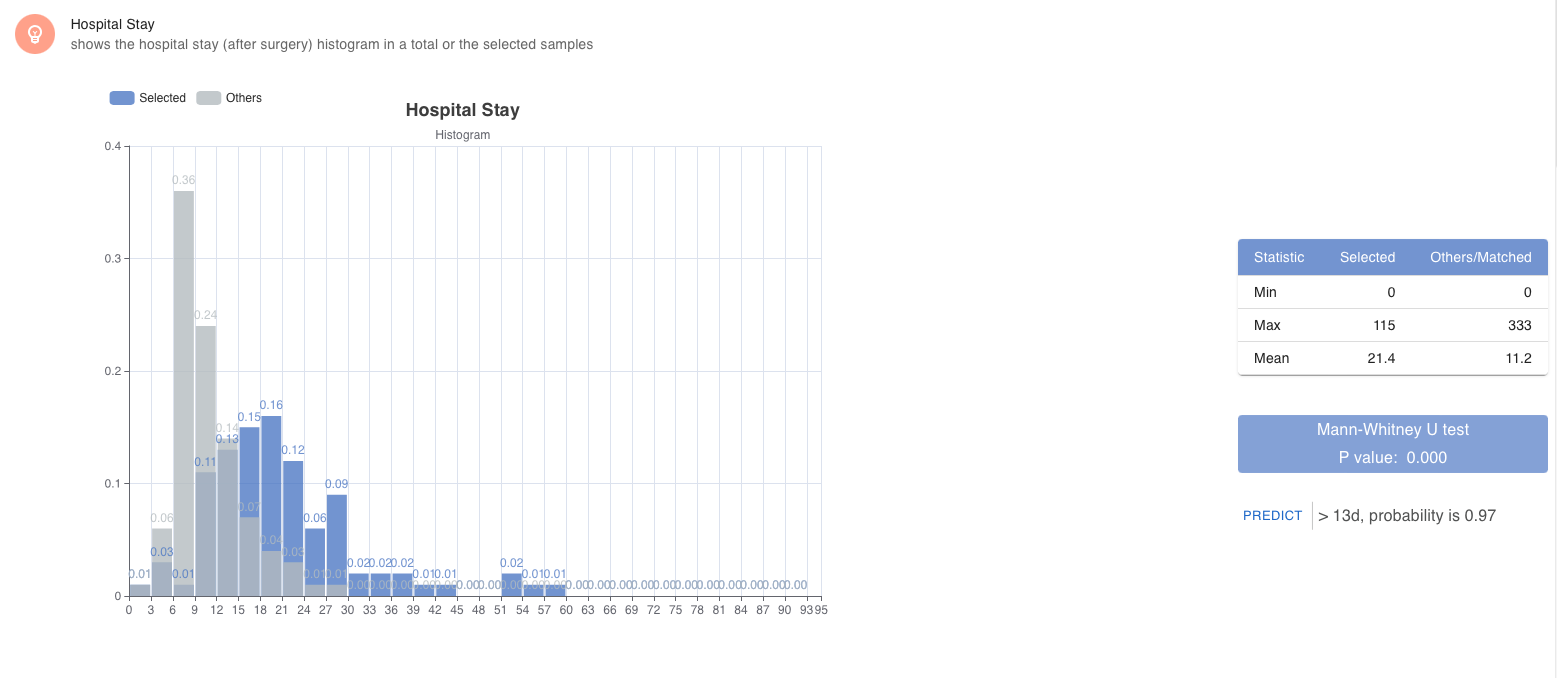


Figure S2 The hospital stay view for the similarity analysis of N.O. 225 case

**Mechanical ventilation time:** From Figure S3, the mechanical ventilation times of 100 patients have several peaks in their distribution. Their mean value is also much higher than the corresponding value of other patients. Predictive values show a 92% likelihood that their duration of mechanical ventilation will exceed 48 hours.


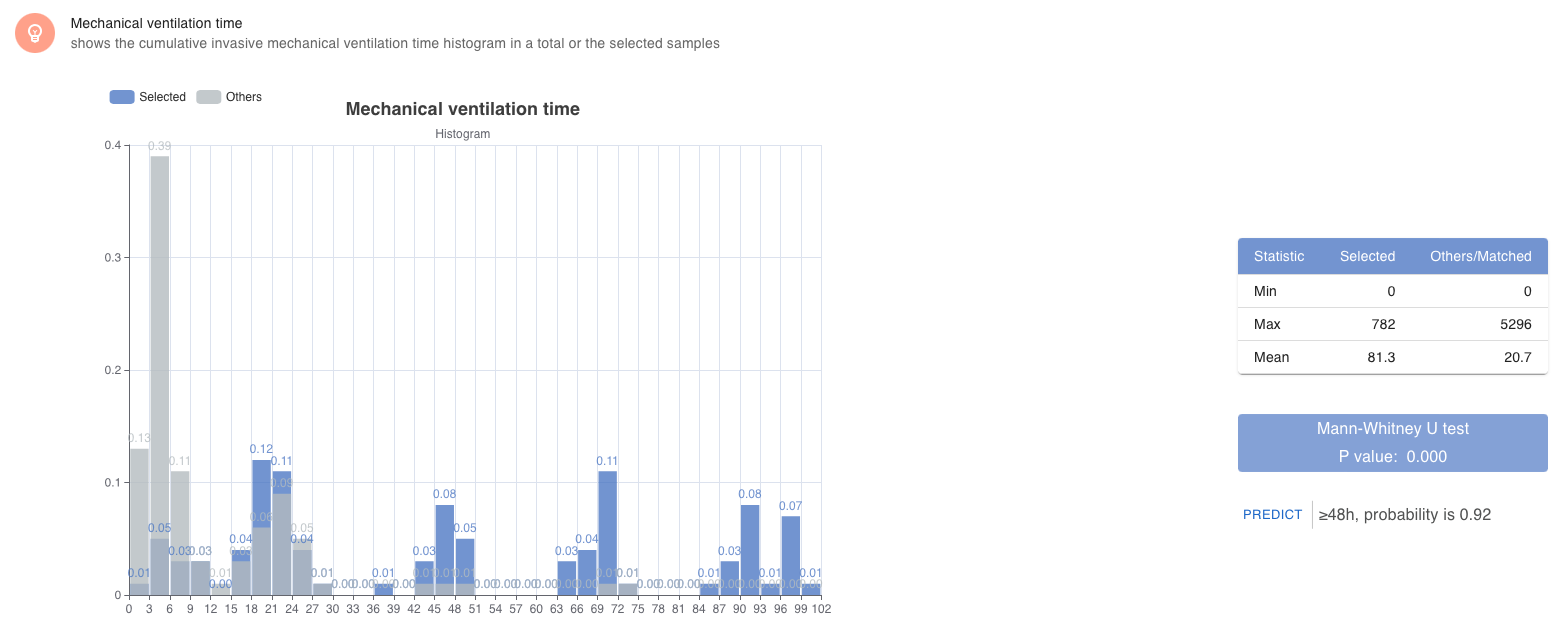


Figure S3 The mechanical ventilation time view for the similarity analysis of N.O. 225 case

**Complication**: The pie chart shown in Figure S4 illustrates that this similar patient group has a higher complication rate, with the predictive value showing a 97% likelihood that this patient will experience a complication.


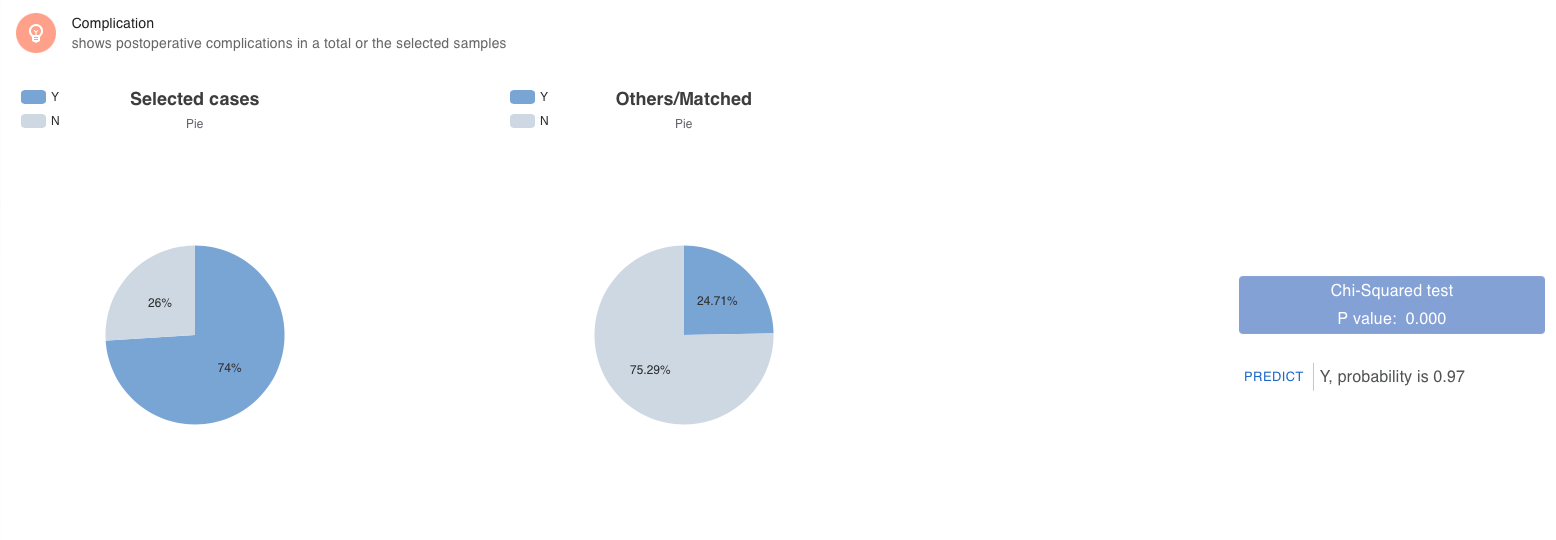


Figure S4 The complication view for the similarity analysis of N.O. 225 case

**Complication Details**: In practice, the user is more concerned about which complications will occur. Therefore, the statistics and lists of hundreds of specific complications in the "Complication Details" view can help users recognize this specific group. As shown in Figure S5, the risk of low cardiac output and delayed sternal closure is doubled for the major complications, and the list below shows that the risk of sepsis is significantly higher for the other complications.


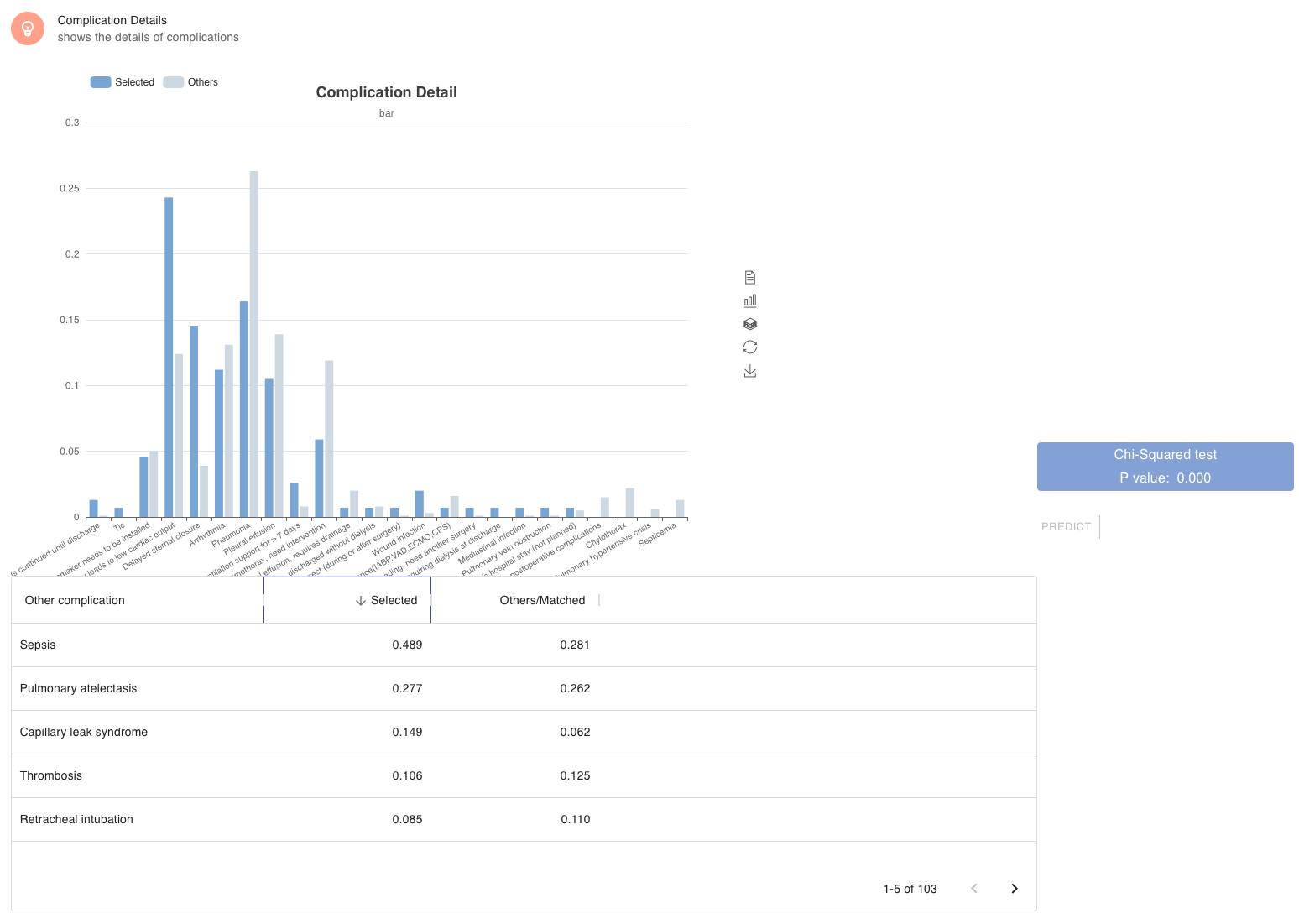


Figure S5 The complication detail view for the similarity analysis of N.O. 225 case

In summary, the user adjusts the parameters to assess the quality of similar groups of patients, and in combination with the statistical metrics of the displayed similar groups of patients, they can form a variety of evidence for decision making for the current patient, thus helping the clinical user to complete the decision-making.

**Table S1**. **Definition of postoperative complications**

| **Complication** | **Definition** |
| --- | --- |
| **Cardiac** |  |
| Cardiac dysfunction resulting in low cardiac output | Low cardiac output state, characterized by use of 3 inotropes and associated with the following: tachycardia, oliguria, decreased skin perfusion, need for increased inotropic support (10% above baseline at admission), metabolic acidosis, widened Arterial – Venous oxygen saturation, need to open the chest, or need for mechanical support. |
| Pulmonary hypertension (PA pressure > systemic pressure) | Clinically significant elevation of pulmonary arterial pressure, requiring intervention, with the pulmonary arterial pressure being greater than the systemic arterial pressure (supra-systemic pulmonary arterial pressure) |
| Pericardial effusion, Requiring drainage | Abnormal accumulation of fluid in the pericardial space, Requiring drainage, By any technique |
| Pulmonary vein obstruction | Clinically significant stenosis or obstruction of pulmonary veins. Typically diagnosed by echocardiography or cardiac catheterization, this may present with or without symptoms |
| Endocarditis-postprocedural infective endocarditis | An infection of the endocardial surface of the heart, which may include one or more heart valves, the mural endocardium, or a septal defect |
| [Cardiac arrest](javascript:;) (Intraoperative or Postoperative) | Cessation of effective cardiac mechanical function necessitating cardiopulmonary resuscitation, developed during or after operation |
| **Arrhythmia** |  |
| Arrhythmia | Any cardiac rhythm other than Normal Sinus Rhythm |
| Arrhythmia necessitating pacemaker, Temporary pacemaker | Implantation and utilization of a temporary pacemaker for treatment of any arrhythmia including heart block (atrioventricular [AV] heart block) |
| **Neurologic** |  |
| Neurological deficit persisting at discharge | Newly recognized and/or newly acquired deficit of neurologic function leading to inpatient referral, therapy, or intervention not otherwise practiced for a similarly unaffected inpatient, With a persisting neurologic deficit present at hospital discharge |
| Paralyzed diaphragm (possible phrenic nerve injury) | Presence of elevated hemi-diaphragm(s) on chest radiograph in conjunction with evidence of weak, immobile, or paradoxical movement assessed by ultrasound or fluoroscopy |
| Seizure | The clinical and/ or electroencephalographic recognition of epileptiform activity. |
| **Pulmonary** |  |
| Pneumonia | A respiratory disease characterized by inflammation of the lung parenchyma (including alveolar spaces and interstitial tissue), most commonly caused by infection |
| Pleural effusion, Requiring drainage | Abnormal accumulation of fluid in the pleural space, Requiring drainage, By any technique. |
| Pneumothorax, Requiring drainage | A collection of gas in the pleural space resulting in collapse of the lung on the affected side. Requiring drainage by chest tube or thoracocentesis |
| Postoperative respiratory insufficiency requiring mechanical ventilatory support >7 days | Respiratory Insufficiency requiring mechanical ventilatory support from surgery or procedure to greater than 7 days postoperatively |
| Chylothorax | Presence of lymphatic fluid in the pleural space, commonly secondary to leakage from the thoracic duct or one of its main tributaries. Thoracocentesis is the gold standard for diagnosis and generally reveals a predominance of lymphocytes and/or a triglyceride level greater than 110 mg/dL |
| Respiratory failure, Requiring tracheostomy | Failure to wean from mechanical ventilation necessitating the creation of a surgical airway |
| **Renal** |  |
| Acute renal failure requiring temporary dialysis with the need for dialysis not present at hospital discharge | New onset oliguria with sustained urine output<0.5 cc/kg/hr for 24 hours and creatinine>1.5 times upper limits of normal for age (or twice the most recent values), with eventual need for dialysis or hemofiltration. The patient does not require dialysis at the time of hospital discharge or death in the hospital |
| **Infectious** |  |
| Sepsis | Evidence of serious infection accompanied by a deleterious systemic response. The diagnosis of sepsis-1 requires the presence of a Systemic Inflammatory Response Syndrome (SIRS) resulting from a proven infection. The diagnosis of sepsis-3 requires infected patients with a Sequential Organ Failure Assessment (SOFA) score≥2 |
| **Wound** |  |
| Wound infection-Mediastinitis | The diagnosis of mediastinitis must meet one of the following criteria: 1: Organisms cultured from mediastinal tissue or fluid that is obtained during a surgical operation or by needle aspiration. 2: Histopathologic examination or visual evidence of mediastinitis seen during a surgical operation. 3: Patient has at least one of the following numbered signs or symptoms with no other recognized cause: 1) fever, 2) chest pain, or 3) sternal instability AND at least one of the following numbered features: 1) purulent mediastinal drainage, 2) organisms cultured from mediastinal blood, drainage or tissue, or 3) widening of the cardio-mediastinal silhouette. 4: Patient≤1 year has at least one of the following numbered signs or symptoms with no other recognized cause: 1) fever, 2) hypothermia, 3) apnea, 4) bradycardia, or 5) sternal instability AND at least one of the following numbered features: 1) purulent mediastinal discharge, 2) organisms cultured from mediastinal blood, drainage or tissue, or 3) widening of the cardio-mediastinal silhouette. |
| Wound infection | Erythema, possible induration and possible fluctuance of a surgical wound with possible drainage and possible tissue separation. Though wound cultures may be positive, this is not an absolute requirement for establishing this clinical diagnosis. |
| **Operative/Procedural** |  |
| Sternum left open | Sternum was left open postoperatively (i.e. planned or unplanned). The goal is for delayed sternotomy closure |
| Mechanical circulatory support IABP, VAD, ECMO, CPS | Institution of intraoperative mechanical support, any type, for resuscitation/CPR or support |
| Reoperation during this admission (unplanned reoperation) | Any additional unplanned operation prior to discharge |
| Bleeding, Requiring reoperation | Postoperative bleeding requiring reoperation |
| **Other** |  |
| Other complication | Any complication not otherwise specified in this list |
| **Operative mortality** |  |

## Table S2 Features used to measure patient similarity

| **Quantitative echocardiographic indicators** | |
| --- | --- |
| Left ventricle (4) | LV diameter, LVEF, interventricular septal thickness, LV posterior wall thickness |
| Left atrium (1) | LA diameter |
| Defects (9) | ASD size, PFO size, VSD size, V_p_ at VSD, ΔP at VSD, PDA size, V_p_ at ductus arteriosus, ΔP at arterial ductal orifice, APW size |
| Pericardial effusion (5) | Anechoic dark area of cardiac apex, LV lateral wall, LV posterior wall, RV anterior wall, and RV posterior wall |
| Semilunar valve (6) | Diameter, V_p_, and ΔP of PV; diameter, V_p_, and ΔP of AV |
| Atrioventricular valve (6) | Diameter, V_p_, and ΔP of TV; diameter, V_p_, and ΔP of MV |
| Aorta (13) | Diameter, V_p_, and ΔP of AO; diameter, V_p_, and ΔP of descending aortic arch; diameter, V_p_, and ΔP of descending aortic; diameter, V_p_, and ΔP of ascending aorta; aortic ride rate |
| Coronary artery (6) | Diameter of LCA, RCA, proximal RCA, distal RCA, left anterior descending branch, and circumflex |
| Pulmonary artery (10) | Pulmonary arterial pressure; diameter, V_p_, and ΔP of LPA, RPA and MPA |
| Ventricular outflow tract (6) | Diameter, V_p_, and ΔP of LVOT and RVOT |
| **CHD diagnosis based on echocardiogram** | |
| CHD diagnosis list | ASD, VSD, PDA, PFO, TOF …… |
| **Patient’s preoperative features** | |
| Preoperative clinical feature (5) | Age, gender, weight, height, preoperative oxygen saturation of right upper limb |
| **Surgical features** | |
| Surgery feature (3) | Surgery time, CPB time, aortic cross-clamping time |

LV left ventricle, LVEF left ventricular ejection fraction, LA left atrium, VSD ventricular septal defect, ASD atrial septal defect, PFO patent foramen ovale, PDA patent ductus arteriosus, APW aortopulmonary window, V_p_ peak velocity, ΔP differential pressure, PV pulmonary valve, AV aortic valve, AO aorta, TV tricuspid valve, MV mitral valve, LCA left coronary artery, RCA right coronary artery, LPA left pulmonary artery, RPA right pulmonary artery, MPA main pulmonary artery, LVOT left ventricular outflow tract, RVOT right ventricular outflow tract, TOF tetralogy of Fallot, CPB cardiopulmonary bypass

**Table S3 Echocardiographic indicators used in different calculations.**

| **Normal and Zscore** | | **Ratio** | |
| --- | --- | --- | --- |
| **Dimension** | **Indicator** | **Dimension** | **Indicator** |
| Left ventricle (4) | LV diameter, LVEF, interventricular septal thickness, LV posterior wall thickness | VSD (9) | VSD size, ΔP at VSD, V_p_ at VSD, size/ V_p_ at VSD, V_p_ of AO/ V_p_ at VSD, VSD size/diameter of AO, V_p_ of TV/ V_p_ at VSD, ΔP at VSD/LVEF, LVEF/ V_p_ at VSD |
| Left atrium (1) | LA diameter | ASD (4) | ASD size, V_p_ of MPA/ ASD size, ΔP of TV/ASD size, ΔP of PV/ASD size |
| Defects (9) | ASD size, PFO size, VSD size, V_p_ at VSD, ΔP at VSD, PDA size, V_p_ at ductus arteriosus, ΔP at arterial ductal orifice, APW size | PDA (3) | PDA size/ΔP at ductus arteriosus, V_p_ of AO/ΔP at ductus arteriosus, V_p_ of AO/ V_p_ at ductus arteriosus |
| Pericardial effusion (5) | Anechoic dark area of cardiac apex, LV lateral wall, LV posterior wall, RV anterior wall, and RV posterior wall | APW (1) | APW size |
| Semilunar valve (6) | Diameter, V_p_, and ΔP of PV; diameter, V_p_, and ΔP of AV | Aortic straddle (1) | Aortic ride rate |
| Atrioventricular valve (6) | Diameter, V_p_, and ΔP of TV; diameter, V_p_, and ΔP of MV | AO (3) | Diameter, V_p_, diameter/ V_p_ of AO, |
| Aorta (13) | Diameter, V_p_, and ΔP of AO; diameter, V_p_, and ΔP of descending aortic arch; diameter, V_p_, and ΔP of descending aortic; diameter, V_p_, and ΔP of ascending aorta; aortic ride rate | Left heart (3) | LA diameter, LV diameter, LVEF, LVEF/AO diameter |
| Coronary artery (6) | Diameter of LCA, RCA, proximal RCA, distal RCA, left anterior descending branch, and circumflex | TV (4) | V_p_ and ΔP of TV, ΔP of TV/ V_p_ of AO, V_p_ of AO/ V_p_ of TV |
| Pulmonary artery (10) | Pulmonary arterial pressure; diameter, V_p_, and ΔP of LPA, RPA and MPA | PA (9) | Diameter and V_p_ of PA, PA pressure/ V_p_ of AO, diameter of PA/diameter of LV, diameter, V_p_, and ΔP of PV, V_p_/ diameter of PV, V_p_ of AO/ V_p_ of PV |
| Ventricular outflow tract (6) | Diameter, V_p_, and ΔP of LVOT and RVOT |  |  |

LV left ventricle, LVEF left ventricular ejection fraction, LA left atrium, VSD ventricular septal defect, ASD atrial septal defect, PFO patent foramen ovale, PDA patent ductus arteriosus, APW aortopulmonary window, V_p_ peak velocity, ΔP differential pressure, PV pulmonary valve, AV aortic valve, AO aorta, TV tricuspid valve, MV mitral valve, LCA left coronary artery, RCA right coronary artery, LPA left pulmonary artery, RPA right pulmonary artery, MPA main pulmonary artery, LVOT left ventricular outflow tract, RVOT right ventricular outflow tract, APW aortopulmonary window
